# Integrated analysis of muscle lncRNA and mRNA of Chinese indigenous breed Ningxiang pig in four developmental stages

**DOI:** 10.3389/fvets.2024.1465389

**Published:** 2024-10-21

**Authors:** Wenwu Chen, Fang Yang, Sui Liufu, Zhi Li, Yan Gong, Haiming Ma

**Affiliations:** ^1^College of Animal Science and Technology, Hunan Agricultural University, Changsha, Hunan, China; ^2^Key Laboratory of Livestock and Poultry Resources Evaluation and Utilization, Ministry of Agriculture and Rural Affairs, Changsha, China; ^3^Yuelushan Laboratory, Changsha, China; ^4^Hunan Key Laboratory for Conservation and Utilization of Biological Resources in the Nanyue Mountainous Region, Hengyang Normal University, Hengyang, China

**Keywords:** Ningxiang pig, muscle, transcriptome, lncRNA, mRNA

## Abstract

Meat and its derivatives serve as crucial sources of protein, vitamins, minerals, and other essential nutrients for humans. Pork stands as China’s primary animal-derived food product consumed widely across diverse dietary structures; evaluating intramuscular fat content becomes pivotal in assessing its quality standards. Nonetheless, the intricate molecular mechanisms governing intramuscular fat deposition remain elusive. Our study utilized sequencing technology to scrutinize longitudinal development stages within Ningxiang pig’s longest dorsal muscles aiming to unravel these underlying mechanisms. In three distinct comparisons (30d vs. 90d, 90d vs. 150d and 150d vs. 210d) there were 578, 1,000 and 3,238 differentially expressed mRNA, along with 16, 158 and 85 lncRNAs were identified. STEM analysis unveiled six enriched model profiles for lncRNAs while seven such profiles emerged for mRNAs; notably, multiple shared model profiles existed between both RNA types. Enriched analysis highlighted numerous genes from mRNA profile8 and lncRNA profile7 significantly associated with pathways linked to fat deposition. Weight Gene Co-Expression Network Analysis (WGCNA) revealed that differential expression modules (DMEs) & differential expression lncRNAs primarily clustered within cyan, dark slate blue and pale turquoise modules. Furthermore, target genes PKD2 (MSTRG21592.MTRSG8859 and MTRSG18175), COL5A1 (MTRSG9969 and MTRSG180) and SOX13 (MTRSG21592 and MTRSG9088) as core components all intricately tied into processes related to fat deposition. This study lays the groundwork for deeper exploration into the molecular mechanisms underlying LDM fat deposition traits, and it also presents candidate genes for future molecular marker-assisted breeding.

## 1 Introduction

As the primary source of human meat on Earth, domestic pigs contribute 40% of global meat consumption (1). Geographical analysis indicates that pig farming was widespread across most regions of the world, with Asia leading with a 60% share (2). According to statistics from FAO Statistical Databases (FAOSTAT: FAO Statistical Databases (FAO, 2023).), in 2023, China’s pig inventory accounted for 58.16% of global pig inventory. As a major producer and consumer of pigs, the quality of pork in China is crucial. The Ningxiang (NX) pig, a renowned local breed, is cherished by consumers for its exceptional meat quality and flavor. Research has shown that the intramuscular fat (IMF) content in NX pigs stands at 5%, significantly higher than the 2% found in commercial pigs (3). Furthermore, indicators such as tenderness and water holding capacity of NX pigs are notably superior to those of commercial pigs (4). However, compared to breeds like Duroc, the growth cycle of NX pigs is longer, leading to higher feeding costs. The skeletal muscles of pigs are primarily characterized by hyperplasia and hypertrophy. Following birth, muscle fiber cells are fixed in structure allowing only for the hypertrophy of these cells to increase muscle mass as is also the case with adipocytes (5). Therefore an in-depth exploration into skeletal muscles is crucial for understanding reasons behind high IMF content in NX pigs.

Long non-coding RNAs (lncRNAs) are prevalent in animals and lacks the capacity for coding. Initially, they were thought to disrupt translation, but recent advances in transcription and molecular biology have revealed their crucial role in various biological processes, including gene imprinting (6), chromatin remodeling (7), splicing regulation (8), mRNA degradation and translation (9). Research indicates that the lncRNA Has2os consistently increases during skeletal muscle differentiation. However, its knockout inhibits differentiation. Experiments further revealed that Has2os influences muscle cell differentiation by modulating the JNK/MAPK pathway (10). Some lncRNAs regulate muscle development by collaborating with miRNAs and exerting a competitive inhibitory effect. For example, miR-101 and lncRNA MALAT1 regulate muscle development via the Mef2A-p38/MAPK signaling pathway (11). Furthermore, lncRNAs not only play a regulatory role in the growth and differentiation of muscle cells but also partially relieve the binding of miRNA to mRNA through competing endogenous RNA (ceRNA), thereby regulating adipocyte differentiation. AI504432 can reduce the inhibitory effect of miRNA on the Fasn gene by competitively binding to miR-1a-3p, thereby promoting adipocyte differentiation (12). Therefore, lncRNAs have regulatory effects on fat and muscle cells.

Drawing from our prior research, it has been established that the types of muscle fibers and the growth and development of NX pigs’ muscles are regulated by various non-coding RNAs (13), including lncRNA, across different developmental stages (14). Additionally, lncRNAs in the liver and subcutaneous adipose tissue at various stages were also investigated (14). Consequently, this study primarily analyzes the IMF in the LDM of NX pigs at different developmental stages, laying a foundation for understanding the mechanisms underlying IMF variations.

## 2 Materials and methods

### 2.1 Experimental animals and sampling

This experiment utilized half-sib animals, born to the same male parent and four female parents under identical rearing conditions. Males were castrated at ages 30, 90, 150, and 210 day and slaughtered at these specific ages to ensure that all pigs slaughtered at a given time point were full siblings. Each time point included three slaughtered animals, totaling twelve in the study. After slaughter, samples of the dorsal muscle from the same region were frozen in liquid nitrogen and stored at −80°C. This study was approved by Use Committee of Hunan Agricultural University, Changsha city, Hunan Province, China and Institutional Animal Care under approval number 2021–13.

### 2.2 RNA library preparation and transcriptome sequencing

The total RNA from tissue samples was then checked by the Nanodrop2000 (NanoDrop Technologies, Wilmington, DE, United States) for extracted RNA concentration and purity, followed by RNA integrity detection using agarose gel electrophoresis and RIN values determined using Agilent2100 (Agilent Technologies, Santa Clara, CA, United States). The requirements for a single library construction are total RNA of 5 ug, concentration of 250 ng/uL, OD 260/280 between 1.8 and 2.2. The Ribo Zero Magnetic kit (Epicentre, Madison, WI, United States) is employed to eliminate ribosomal RNA. Subsequently, the TruSeqTM Stranded Total RNA Library Prep Kit is utilized to construct strand-specific libraries for the detection of lncRNA and mRNA. Sequencing of strand-specific libraries and small RNA libraries was conducted on the HiSeq 4,000 platform using PE150 and SE50, respectively. The deep sequencing was executed by Majorbio Biopharmaceutical Technology Co., Ltd. (Shanghai, China).

### 2.3 Transcripts assembly

Due to the presence of many interfering sequences and low-quality reads in the original sequencing data, we used Sickle^1^ and SeqPrep^2^ to filter out the original sequence information for sequencing, removing uninserted fragments and fragments carrying connecting sub sequences, and screening out reads with N values greater than 10%. Align and locate the obtained clean reads with the reference genome of NX pigs, while cutting the large reads into smaller segments aligned with the reference genome.

### 2.4 LncRNA identification

Select RNA with a length greater than 200 bp that does not overlap with known mRNA, and then score the selected RNA using CNCI 2.0 (15). RNA with a score of<0 will be excluded, and the remaining RNA will be scored using CPC 0.9-r2 (16). RNA with a score of<1 will be excluded, and the remaining RNA will be considered lncRNA.

### 2.5 Analysis of differentially expressed genes and differentially expressed lncRNAs

StringTie was used to display the expression levels of mRNA and lncRN by calculating the FPKM of each sample. Then use R package edge R to calculate the mRNA and lncRNA differentially expressed with log2 (fold change) <−1 or log2 (fold change) >1 and statistical significance (*p* < 0.005). To investigate the enrichment of differentially expressed genes in various closed groups, we employed Gene Ontology (GO) and the Kyoto Encyclopedia of Genes and Genomes (KEGG).

### 2.6 Prediction of target genes of lncRNAs and network analysis

LncRNA may bind to neighboring target genes through cis interactions, thereby regulating gene expression. Therefore, we used a Python script to select the target genes of lncRNA, and functional analysis was performed on the upstream and downstream coding genes of lncRNA using BLAST2GSO (17). The significantly expressed *p* < 0.005. TargetScan and miRanda were used to predict the association between lncRNA and mRNA, and Pearson correlation coefficients were used to evaluate the association between lncRNA and mRNA. Finally, Cyto-scape software was used to visualize the regulatory network.

### 2.7 Short time-series expression miner (STEM) analysis

STEM was utilized to categorize the genes and lncRNA expression patterns. Following the importation of mRNA and lncRNA data from four distinct time periods, they will be organized into clusters based on genes exhibiting similar expression trends, subsequently being assigned to the same module. The colored background indicates a significant trend (adjusted *p* ≤ 0.05 by Bonferroni correction), while the gray one indicates no significant trend. Modules of the same color demonstrate similar trends in gene expression.

### 2.8 Weighted correlation network analysis (WGCNA)

Initially, a gene co-expression network is established. The Pearson correlation coefficient is then calculated for the expression patterns of pairs of genes. Using this correlation coefficient, a gene network is constructed. Subsequently, genes with closely related expression patterns are grouped into modules based on a defined threshold (*β* value). Feature values are assigned to these modules, and GO enrichment analysis is conducted on the individual genes within them.

### 2.9 Predicted mRNA and lncRNA validation by qRT-PCR

Using the Animal Total RNA Kit (Tiangen, China) to extract total RNA from muscle of NX pigs, Prime-script RT Master Kit (Thermo Scientific, United States) and random primers were used to reverse the total RNA into cDNA. Then, Primer 5.0 was used to design primers, and all reactions were carried out three times for each sample. The relative expression levels of lncRNAs and mRNAs were was calculated via the comparative CT method (2^–△△CT^). Please refer to previous papers for details (18).

### 2.10 Statistical analysis

The unpaired two-tailed T-test was utilized to assess the differential expression of mRNA and lncRNA at 2 distinct time points; data were showed as ± SEM.

## 3 Results

### 3.1 Classification and identification of lncRNA in NX pig LDM

**Figure 1:**
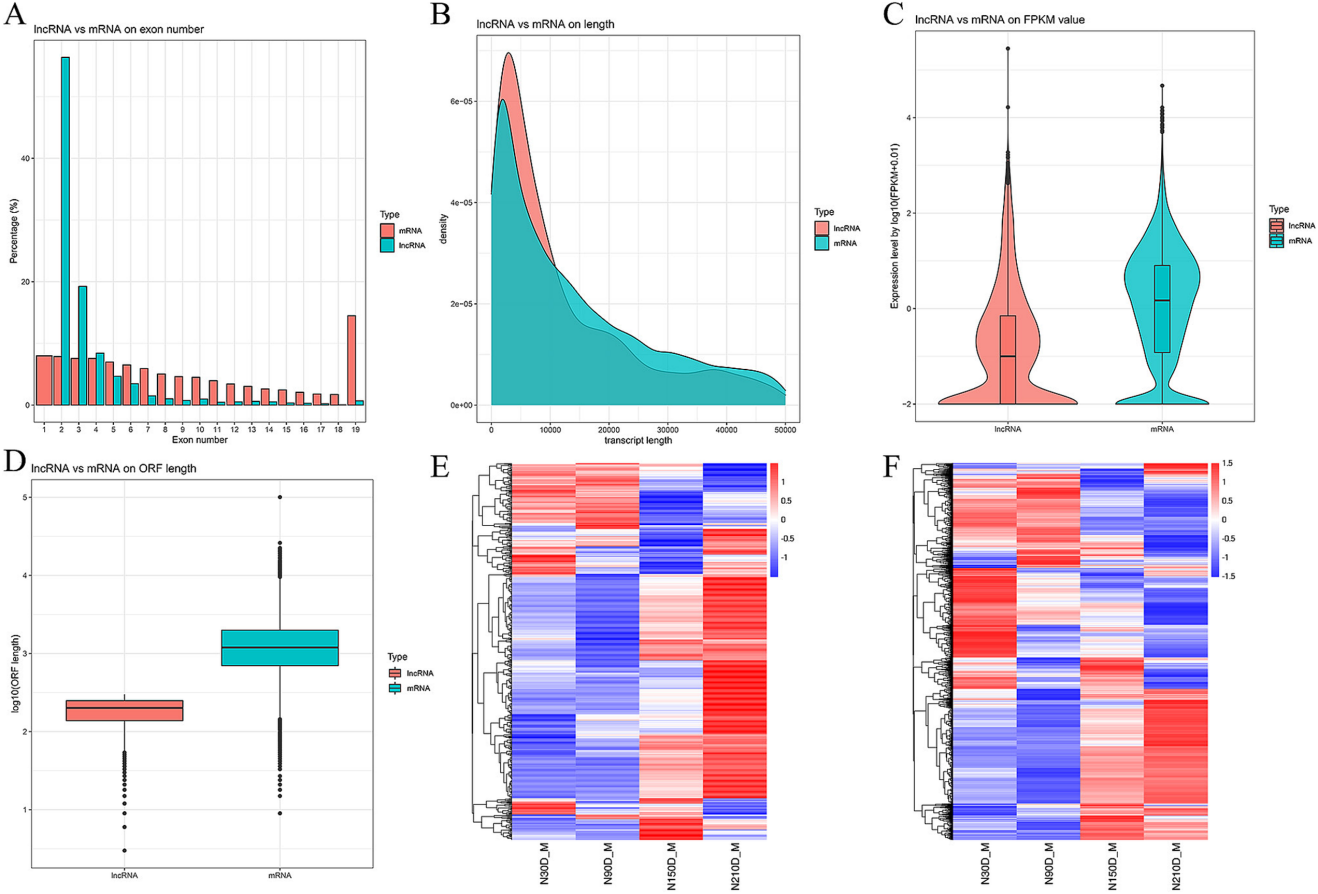
Comparison between lncRNA and mRNA on exon number **(A)**, length **(B)**, fragments per kilobase of exon per million mapped reads (FRKM) value **(C)**, and open reading frame (ORF) length **(D)**. Heat maps depicting differentially expressed mRNAs **(E)** and lncRNAs **(F)** along with their expression modes.

The LDM of NX pigs at four different developmental stages was detected to be 128,342,221, 97,849,492, 105,862,852, and 11,0513,258 clean reads with Q30 exceeding 95.24% (Table 1). In total, 3,170 novel RNAs were identified, which comprised of 1,443 intergenic, 47 sense intro overlapping, 828 antisense, 817 sense exons and 35 bidirectional (Supplementary material S2). The majority of lncRNAs consist of 2 or 3 exons (Figure 1A). Additionally, lncRNAs are generally shorter in length and exhibit lower expression levels compared to mRNA (Figures 1B,C). Furthermore, data pertaining to open reading frames reveals that lncRNAs typically possess shorter open reading frames than mRNA (Figure 1D). Analysis of the mRNA expression profiles reveals a similarity in expression patterns at 30d and 90d, with a majority of mRNA exhibiting low levels of expression. However, there is a notable increase in high-expression genes during the middle and later stages (Figure 1E). In contrast, lncRNA demonstrates high expression at 30d and 90d but relatively weak expression at 210d, suggesting a potential attenuation of regulatory control in the later stages of development (Figure 1F).

**Table 1:** Statistic of raw and clean reads in LDM.

Sample	Raw reads	Raw bases	Clean reads	Clean bases	Error rate (%)	Q20 (%)	Q30 (%)
N30D-1	125,643,820.00	18,972,216,820.00	122,988,528.00	16,416,455,345.00	0.02	98.34	95.45
N30D-2	131,506,100.00	19,857,421,100.00	129,299,096.00	17,379,678,801.00	0.02	98.42	95.54
N30D-3	136,491,376.00	20,610,197,776.00	132,739,040.00	17,179,099,621.00	0.02	98.32	95.35
N90D-1	95,652,458.00	14,443,521,158.00	92,263,860.00	12,115,008,995.00	0.02	98.37	95.60
N90D-2	106,914,554.00	16,144,097,654.00	103,813,926.00	13,538,546,126.00	0.02	98.25	95.29
N90D-3	100,365,882.00	15,155,248,182.00	97,470,692.00	12,848,257,336.00	0.02	98.21	95.28
N150D-1	105,975,290.00	16,002,268,790.00	104,317,724.00	14,219,362,833.00	0.02	98.35	95.32
N150D-2	109,666,184.00	16,559,593,784.00	107,345,576.00	14,444,210,597.00	0.02	98.46	95.58
N150D-3	107,934,054.00	16,298,042,154.00	105,925,256.00	14,356,819,599.00	0.02	98.35	95.24
N210D-1	111,363,074.00	16,815,824,174.00	109,677,650.00	14,537,467,677.00	0.02	98.53	95.78
N210D-2	115,451,140.00	17,433,122,140.00	113,851,010.00	15,236,413,428.00	0.02	98.56	95.75
N210D-3	109,831,316.00	16,584,528,716.00	108,011,116.00	14,410,474,601.00	0.02	98.44	95.44

### 3.2 Analysis of mRNA and lncRNA differentially expressed during closed time periods

**Figure 2:**
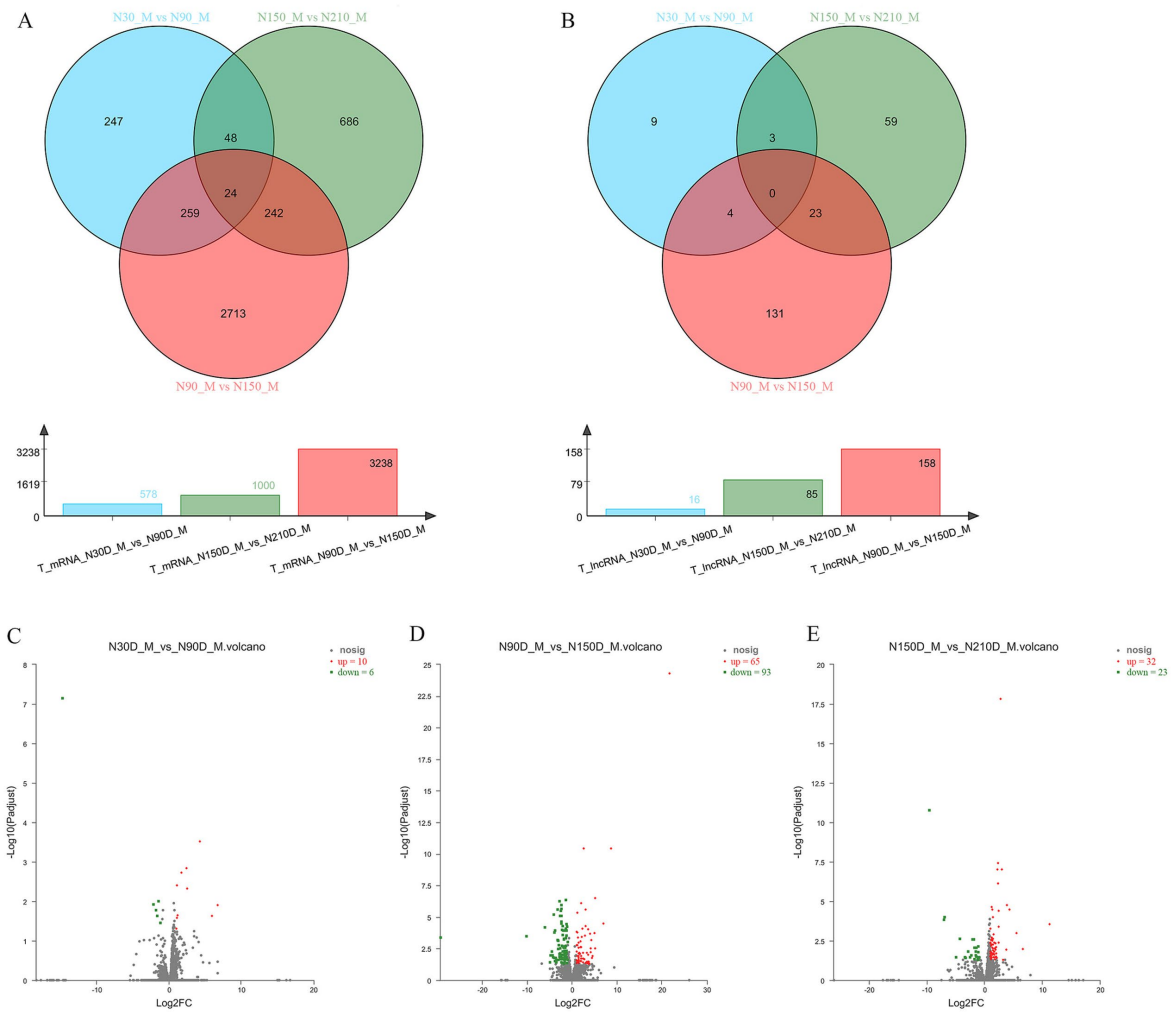
The Venn diagram illustrates the co-expression of mRNA and lncRNA across three distinct groups at four developmental stages, while the bar chart displays the number of co-expressed mRNA and lncRNA in each group **(A,B)**. Volcano plot demonstrating significant up- and down-regulation of genes within three distinct groups **(C–E)**.

To further explore the expression differences of mRNA and lncRNA across various time periods, we conducted pairwise comparisons among four time points, resulting in three closed groups: 30d vs. 90d, 90d vs. 150d, and 150d vs. 210d. Within these three closed groups 578, 1,000 and 3,238 differentially expressed mRNA, along with 16, 158 and 85 lncRNAs were identified (Figures 2A,B).

By comparing the differentially expressed genes within each group, there were 10 upregulated genes and 6 downregulated genes in the group of 30d vs. 90d, 65 upregulated genes and 93 downregulated genes in the group of 90d vs. 150d, and 32 upregulated genes and 23 downregulated genes in the group of 150d vs. 210d (Figures 2C–D; Supplementary materials S2–S4).

### 3.3 Conduct short times series analysis on mRNA and lncRNA across four time periods

**Figure 3:**
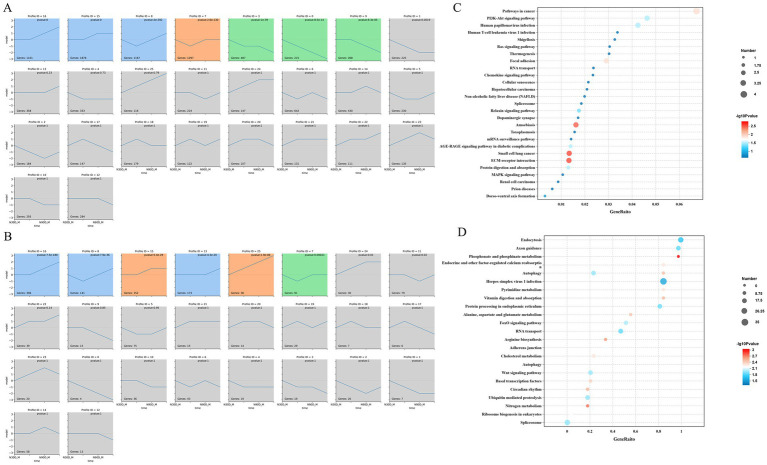
STEM analysis of the mRNA **(A)** and lncRNA **(B)**. Number on the top means module number. The *p*-value in the module indicated the statistically significant *p*-value. Number in the lower-left corner indicate the numbers of mRNAs and lncRNAs in each module. The same color means there belong to same cluster. The enrichment pathway of the mRNA profile 8 **(C)** and lncRNA profile 7 **(D)** were show in the picture.

Through STEM analysis of four developmental stages, all lncRNA in the LDM were categorized into 3 cluster profiles, including 6 enriched model profiles. The expression trends for profiles 16, 15, 25 and 13 generally increased, whereas those for profiles 8 and 7 initially decreased before increasing (Figure 3A). The mRNA in the LDM was divided into 3 cluster profiles, with a total of 7 enriched model profiles. Specifically, the trends for profiles 16 and 15 are upward, whereas for profiles 3, 9 and 0, the trends are gradually decreasing (Figure 3B).

Through enrichment analysis of mRNA from different modules, it was found that mRNA in profile 8 is significantly enriched in pathways such as phosphonate and phosphinate metabolism, Nitrogen metabolism, Wnt signaling pathway, and arginine biosynthesis (Figure 3C). The lncRNA in profile 7 is mainly enriched in signaling pathways such as ECM-receptor interaction, Focal adhesion and PI3K-Akt signaling pathway (Figure 3D).

### 3.4 WGCNA co-expression network analysis of lncRNA and mRNA

**Figure 4:**
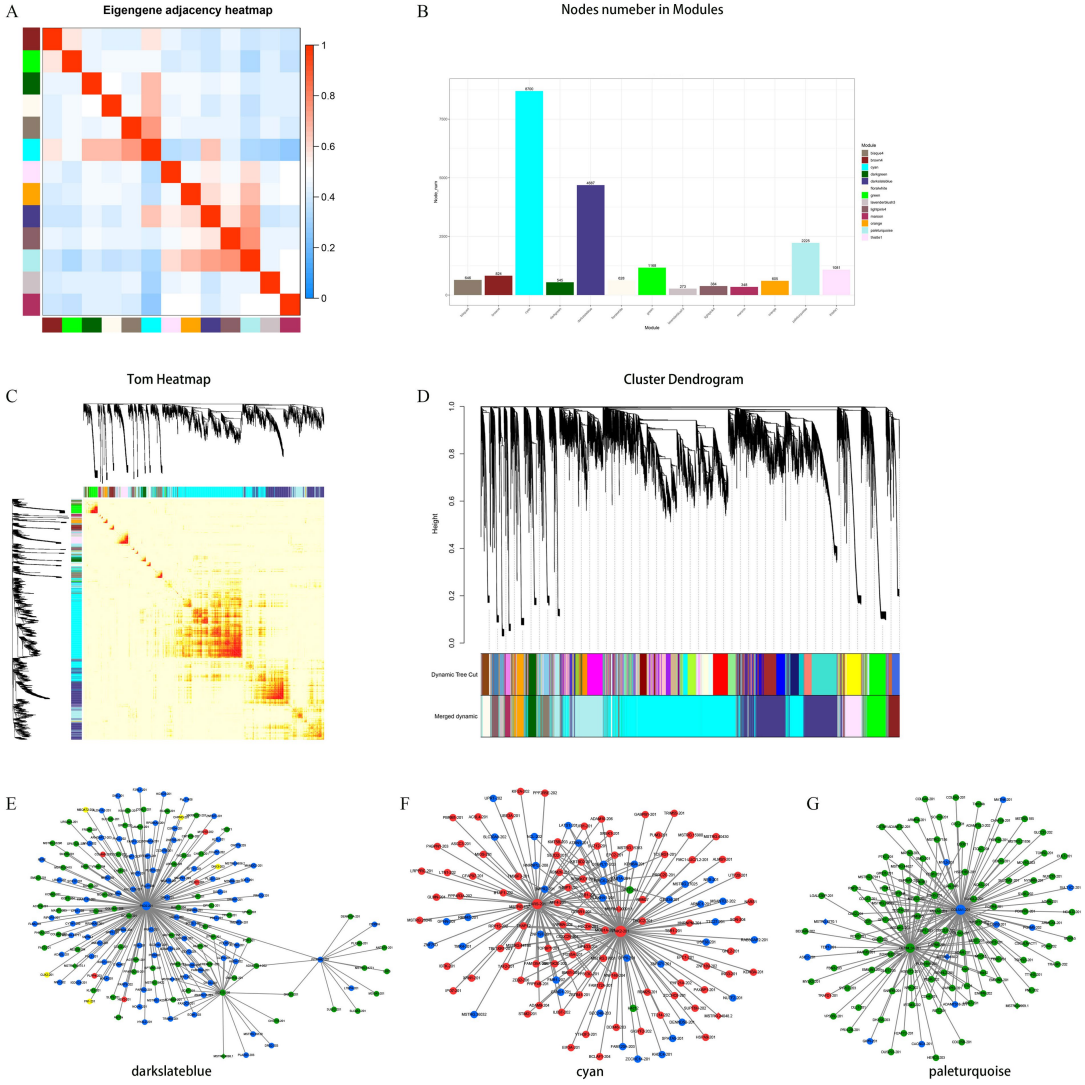
Hierarchical clustering dendrogram of module eigengenes and heatmap of adjacencies **(A,C)**. Number of the lncRNA or mRNA in the 13 modules, the different color means different cluster **(B)**. Hierarchical clustering dendrogram of lncRNA–mRNA co-expression modules. Each branches represents a cluster of lncRNAs or mRNAs. Dynamic tree cut represents original split module, and merged dynamic represents final merge module **(D)**. The darkslateblue represent cellular component module **(E)**, the cyan means biological process module **(F)**, the paleturquoise means molecular function module **(G)**. The up-regulated types are represented in red, while the down-regulated types are depicted in green. Types exhibiting both up- and down-regulation are considered different and are indicated in yellow. Diamonds represent mRNA genes, while rounds represent lncRNA genes.

To explore the potential functional regulatory relationship and mechanism of lncRNA regulatory protein coding genes in the expression process, WGCNA was employed to construct the co-expression network of protein coding genes and lncRNA in the development of muscle tissue in NX pigs. The aim was to investigate the hub gene in different modules and their functions. All samples were available for subsequent analysis under cluster classification. A total of 19,978 mRNA and 2,683 lncRNA obtained by transcription were included in the analysis, while 1,682 lncRNA and 487 mRNA did not meet the requirements and were eliminated. Ultimately, 18,296 mRNA and 2,196 lncRNA were analyzed. These lncRNAs and mRNAs were divided into 13 modules (Figures 4A–C), with a focus on the top three modules (cyan, darkslateblue and paleturquoise) for subsequent analysis (Figure 4D). In the cyan module, genes *PAK2*, *LARS* and *BIRC6* are positioned centrally and considered as hub genes. Within the darkslateblue module, genes *PKD2*, *SOX13* and *PITPNM2* also hold central positions and are regarded as hub genes. Similarly, in the paleturquoise module, genes *COL5A1*, *OLFML2A* and *P3H3* occupy central positions and are recognized as hub genes (Figures 4E–G and Table 2).

**Table 2:** Hub genes and associated lncRNAs in same module.

Hub gene	Module ID	Gene function description	Associated lncRNA
*PAK2*	Cyan	Encodes serine/threonine protein kinases that are involved in regulating the cytoskeleton, cell motility, cell cycle progression, and cells Apoptosis and proliferation.	MSTRG.24048.2, MSTRG.20091.1
*LARS*	Cyan	Encoding leucyl-TRNA synthase, which is present in the cytoplasm as part of the multisynthase complex, interacts with arginine tRNA synthase using the C-terminal structure	MSTRG.20091.1
*BIRC6*	Cyan	Encodes anti-apoptotic proteins that regulate cell apoptosis by regulating caspase and acting as E3 ubiquitin protein ligase	-
*PKD2*	Darkslateblue	Encodes polycystic proteins that regulate the release of calcium ions stored in the endoplasmic reticulum	MSTRG.21592.2, MSTRG.8859.2, MSTRG.18175.1
*SOX13*	Darkslateblue	Encodes the transcription factor SOX13, which binds to DNA at the 5’-AACAAT-3′ site. It can promote the differentiation of brown fat cells	MSTRG.21592.2, MSTRG.9088.1
*PITPNM2*	Darkslateblue	Encodes phosphatidyl inositol transfer protein, catalyzes the transfer of phosphatidyl inositol and phosphatidylcholine between membranes	-
*COL5A1*	Paleturquoise	Encodes Type V collagen, which binds to DNA, heparin sulfate, thrombolin, heparin, and insulin	MSTRG.9969.1, MSTRG.180.2
*OLFML2A*	Paleturquoise	Encoding olfactory protein-like protein 2A	MSTRG.6770.1, MSTRG.180.2
*P3H3*	Paleturquoise	The enzyme prolyl 3-hydroxylase 3, a catalytic complex composed of PLOD1, P3H3, and P3H4, is responsible for catalyzing the hydroxylation of lysine residues in collagen alpha chains. This process is essential for the normal assembly and cross-linking of collagen fibers.	-

### 3.5 RT-qPCR quantification of lncRNA and mRNAs

**Figure 5:**
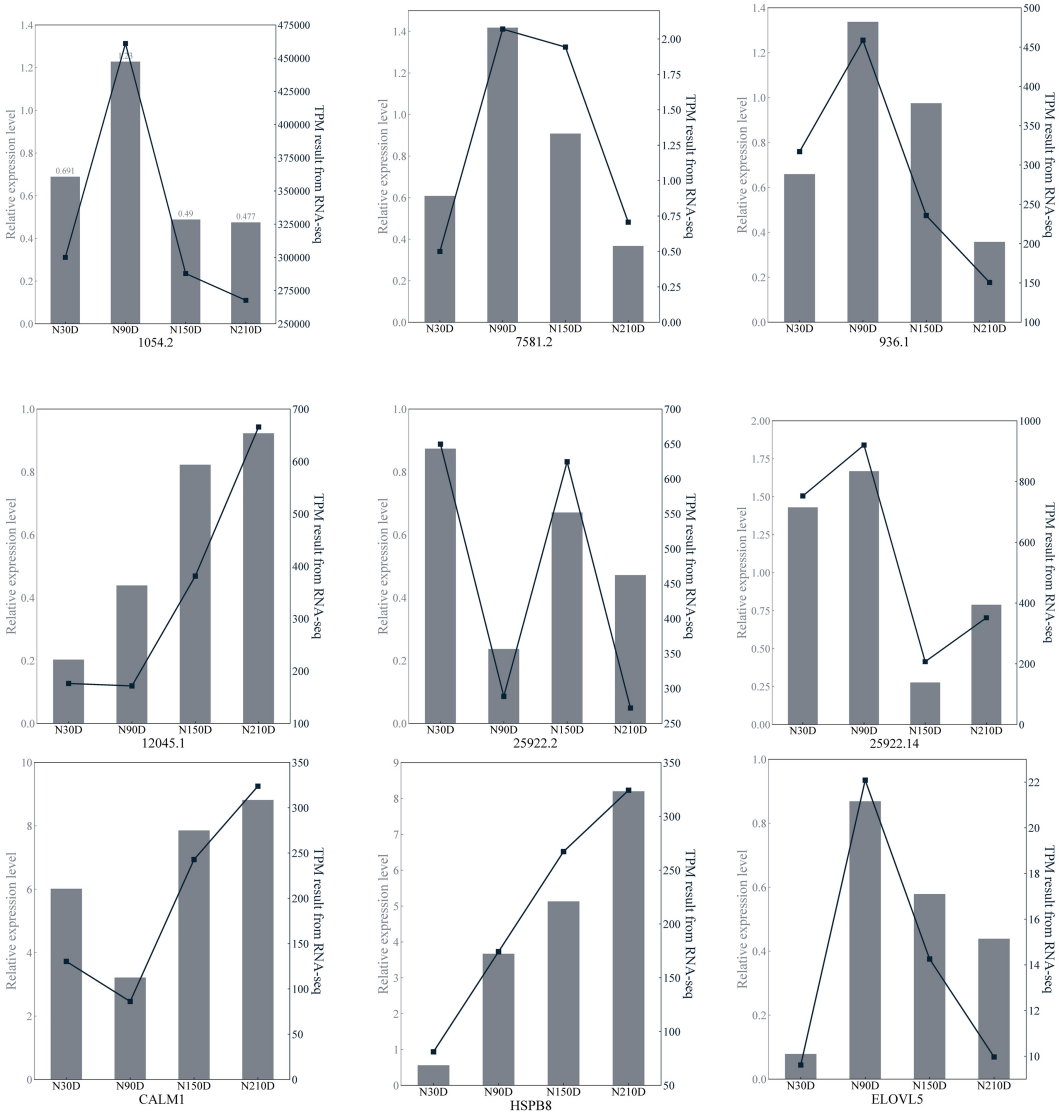
Transcription patterns of MSTRG.1054.2, MSTRG.7581.2, MSTRG.963.1, MSTRG.12045.1, MSTRG.25922.2, MSTRG.25922.14, CLAM1, HSPB8, ELOVL5 compared to expression patterns in the RNA-seq.

Six lncRNAs exhibiting high expression levels were randomly selected at four developmental stages of NX pig muscle tissue (30, 90, 150, 210 days after birth), and quantitative analysis was conducted using RT-qPCR with the primers listed in Table 3. Additionally, three genes including *ELOVL5* associated with fatty acid metabolism were chosen for quantitative analysis using RT-qPCR with the primers specified in Table 4. As depicted in Figure 5, the RNA-seq data and RT-qPCR results showed a high degree of consistency, validating the reliability of the sequencing outcomes.

**Table 3:** Forward and reverse primers of lncRNAs.

Primer	Primer sequence (5′ → 3′)	Tm (°C)	Product length (bp)
MSTRG.25922.14-F	GAGGAGACCGGCGAAGGG	61.7	512
MSTRG.25922.14-R	CTGTCTGAGCGTCGCTTGA	57.3	
MSTRG.25922.2-F	GATTCCGACTTCCATGGCCA	57.4	599
MSTRG.25922.2-R	GTGGCGCAATGAAGGTGAAG	57.4	
MSTRG.12045.1-F	CGCTGAGCTGTTGGGTATGA	57.4	203
MSTRG.12045.1-R	AGCGTTGGGAAGTGCTCTTT	55.4	
MSTRG.963.1-F	TTTTTAGCAGGGGAGCGCAG	57.4	211
MSTRG.963.1-R	GCGATCTGGCTGTGACATCT	57.4	
MSTRG.7581.2-F	GTTCACCCAGAGCTGTACCC	59.5	299
MSTRG.7581.2-R	CCAAGGGGAGGTTCCTTGAC	59.5	
MSTRG.1054.2-F	TGTAGTCCCAGCTACTCGGG	57.4	273
MSTRG.1054.2-R	ACAGGGTCTCGCTATGTTGC	59.5	

**Table 4:** Forward and reverse primers of mRNAs.

ID	Primer	Primer sequence (5′ → 3′)	Tm (°C)	Product length (bp)
*CLAM1*	Gene = Pig.12036-F	ACTGTCATGAGGTCATTGGGTC	57.67	162
	Gene = Pig.12036-R	CTCGCGGATTTCTTCTTCGCTG	59.54	
*ELOVL5*	Gene = Pig.17800-F	CATCCTGCGCAAGAACAACC	57.45	180
	Gene = Pig.17800-R	GGGATGGATGACAGACCGTAG	59.52	
*HSPB8*	Gene = Pig.08614-F	AACATCAAGACCCTGGGCGAT	57.57	151
	Gene = Pig.08614-R	GAGCAAAGGTGTTCATGACGG	57.57	

## 4 Discussion

Muscle growth and development primarily manifest as the growth and hypertrophy of muscle cells, with minimal changes in the number of muscle fibers. Currently, pork serves as the primary source of animal protein for human consumption. The longissimus dorsi muscle is a key component in evaluating pork quality. Studies on pork quality predominantly focus on the characteristics of muscle fiber and IMF, with IMF content showing a positive correlation with meat tenderness, flavor, and juiciness. NX pigs are renowned for their high-quality pork. An increasing body of research suggests that lncRNA plays a significant role in muscle growth and development, IMF deposition, and fatty acid composition. In this study, a substantial number of differentially expressed mRNAs (DEGs) and lncRNAs (DELs) were identified in 4 stages of NX pig muscle tissues: 4816 DEG and 259 DEL (*p* < 0.05). Among the DEGs and DELs, the greatest difference was observed between 150 days vs. 210 days that indicating significant differences in muscle development at 210 days compared to other time points.

KEGG enrichment analysis of DEGs at four time points revealed that numerous upregulated DEGs were associated with immune pathways during the early growth phase of NX pigs. Specifically, upregulated DEGs at 30 vs. 90 days were primarily enriched in immune signaling pathways, including the T cell receptor signaling pathway, Natural killer cell mediated cytotoxicity and Primary immunodeficiency. In the 90 vs. 150 days comparison, upregulated genes were primarily enriched in pathways such as MAPK signaling pathway, IL-17 signaling pathway, NOD-like receptor signaling pathway and TNF signaling pathway (Supplementary materials S5, S6). In the later growth phase, most upregulated DEGs were associated with pathways related to fat accumulation, including the Wnt signaling pathway, cGMP−PKG signaling pathway and MAPK signaling pathway in the 150 vs. 210 days comparison (Supplementary material S7). 10 lncRNAs expressions were found to be involved in the regulation related to lipid metabolism in 30vs90 days. This indicates that 90 day is an important stage for lipid metabolism during the development of NX pigs. Additionally, when compared with 150 days, it was observed that 23 DELs were downregulated at 210 days. Their target genes were enriched in phospholipase activity regulation and lipase activity regulation, suggesting a reduction in the expression level of lncRNAs involved in lipid regulation function at 210 days. Lipase present in the blood promotes triglyceride decomposition to provide fatty acids for triglyceride synthesis and fat deposition promotion. However, as lipase mainly participates in body fat decomposition, a decline in its regulatory function can lead to obesity and further promote fat deposition. At 210 days, reduced lncRNA expression levels regulating lipase activity accelerated fat deposition within muscle tissue.

This study employed STEM analysis to uncover the dynamic expression patterns of lncRNA and mRNA during the development of LDM in NX pigs. The analysis revealed that profiles 16, 8, 15 and 7 exhibited similar expression trends across both omics, suggesting a high degree of correlation between these modules during pig development. Predictions of lncRNA target genes revealed that certain protein-coding genes within profiles 15 and 16 of the mRNA module were also targeted by lncRNA profiles 15 and 16. The target gene of MSTRG.19683.1, MSTRG.20242.2 and MSTRG.860.1 in module 15 is *LEPR*, while the target gene of MSTRG.19683.1, MSTRG.20242.2 and MSTRG.860.1 is *KLF7*; additionally, the gene of MSTRG1058 0.1 in module 16 is *SMGS1*.

The *LEPR* gene was categorized as a type I cytokine receptor, capable of binding with leptin to regulate lipid metabolism in the body (19). Previous research indicates that this gene serves as a genetic marker linked to growth speed and fattening (20). In Suzhong pigs, which are hybridized with foreign breeds in China, high levels of *LEPR* expression were detected in both back fat and the LDM, with significantly elevated expression in high-fat pigs compared to low-fat ones (21); *KLF7* gene is widely present in animals and is a type of regulatory factor related to transcription and metabolism. It is a member of the *SP*/*KLF* transcription factor family and its gene coding sequence exhibits high homology among mammals (22). Induced differentiation of preadipocytes reveals that *KLF7* exerts a negative regulatory effect on these cells. Overexpression of *KLF7* hinders the differentiation of preadipocytes, leading to reduced expression of *PPARγ* and *A-FABP*, whereas overexpression in mature adipocytes has no impact on the expression of *PPARγ* and *A-FABP* (23). In broiler chickens, this gene has also been linked to fat traits such as very low density lipoprotein levels and abdominal fat weight (24). In mice fed a high-fat diet, the gene shows significantly elevated expression. Overexpression of this gene can lead to reduced expression of adiponectin and leptin, as well as hexokinase in skeletal muscle, accelerating energy metabolism and enhancing muscle fat deposition (25);*SMGS1* gene encodes sphingomyelin synthase, a protein composed of 413 amino acids, which shares up to 97% sequence similarity with human proteins and is extensively expressed in the body (26). Sphingomyelin synthase catalyzes the synthesis of sphingomyelin and diglycerides from lecithin and ceramide. Sphingomyelin synthase modulates lipid raft receptors by regulating sphingomyelin synthesis, thereby influencing fatty acid metabolism. Overexpression of *SMGS1* at the cellular level has been observed to enrich the production of triglycerides with polyunsaturated fatty acids (27). Currently, sphingomyelin synthase has emerged as a new target for research in the treatment of arteriosclerosis (28). Based on the STEM decomposition results, the IMF content of LDM in NX pigs is modulated by a variety of lncRNAs during the developmental process and lncRNAs can impact fat deposition and metabolism of NX pigs through diverse metabolic pathways.

To investigate the potential functional regulatory relationship between lncRNA and mRNA during body development, a co-expression network of lncRNA and lncRNA in NX pig muscle tissue development was established to identify the core genes in different modules. The co-expression network analysis revealed *PAK2*, *LARS* and *BIRC6* as the core genes in the cyan module. *PKD2*, *SOX13* and *PITPNM2* were identified as core genes in the darkslateblue module. In the paleturquoise module, the core genes are *COL5A1*, *OLFML2A* and *P3H3* (Figure 4).

*PKD2* (protein kinase D2) serves as an effector for diacylglycerol and protein kinase C (*PKC*), and also acts as a central regulator of nutritional homeostasis (29). It facilitates lipid uptake in the intestine by directly interacting with intestinal cells. Research indicates that *PKD* is activated by free fatty acids (FFAs) and diacylglycerol (DAG) (30) and inhibiting PKD pathways results in heightened energy expenditure in adipocytes (31). Within the mRNA-lncRNA co-expression network of NX pig muscle tissue, this gene was found to be associated with three lncRNAs (MSTRG.21592.2, MSTRG.8859.2 and MSTRG.18175.1) and two protein-encoding genes *ACADL* and *FADS3* which are related to fatty acid metabolism. *ACADL* encodes a long-chain-specific acyl-CoA dehydrogenase, serving as the initial catalyst for releasing energy through mitochondrial fatty acid oxidation. It exhibits activity toward unsaturated acyl-CoA ranging from 6 to 24 carbons in length, with a preference for chains containing 8 to 18 carbons (22). Linkage disequilibrium analysis revealed a significant correlation between porcine *ACADL* polymorphism and IMF content in the LDM (32). The *FADS3* gene facilitates conversion of n-6 polyunsaturated fatty acids (PUFAs) to n-3 PUFAs, thereby enhancing levels of n-3 PUFAs within the body (33).

*COL5A1* (Collagen Type V Alpha 1 Chain) encodes a low-abundance fibrous collagen alpha chain. These fibrous collagen molecules form trimers and can be composed of one or more types of alpha chains. Research has showed that the deletion of the *PLXND1* gene in zebrafish can lead to increased expression of *COL5A1*, which in turn promotes the proliferation and differentiation of adipocytes, resulting in morphological hyperplasia of visceral adipose tissue (34). Additionally, Sheng et al. identified *COL5A1* as a core gene through WGCNA analysis of adipose tissue from cow (35), while Chen et al. also found *COL5A1* to be a core gene through analysis of LDM in pigs, findings consistent with this research (36). MSTRG.9969.1 and MSTRG.180.2 were associated with this gene by co-expression network suggested they will regulate the expression of *COL5A1*. SOX13 (SRY-Box Transcription Factor 13) is a member of the SOX protein family, extensively expressed in body tissues. The protein, containing an HMG domain, can specifically bind to the DNA sequence AACAAT, endowing it with transcription factor activity. Previous research indicates that islet cell antibody 12 (*ICA12*) corresponds to the *SOX13* protein (37). The growth rate of *SOX13* deficient mice is faster than that of normal mice, while inhibiting the differentiation of αβ T cells and the Wnt signaling pathway (38). By WGCNA analysis there were MSTRG.21592.2 and MSTRG.9088.1 are associated with *SOX13*. The aforementioned studies indicate that, in the developmental process of NX pigs, lncRNA may influence the IMF deposition in LDM by regulating core genes.

## 5 Conclusion

In this study unveiled the expression profiles of mRNA and lncRNA at four time points during the development of LDM in NX pigs. STEM analysis revealed multiple expression modules with similar trends between DEGs and DELs at the four developmental time points, while enrichment analysis indicated that numerous mRNAs in profile8 were enriched in pathways associated with fat development, such as the Wnt signaling pathway. Additionally, several lncRNAs were also found to be enriched in PI3K-Atk and other signaling pathways related to fat development. WGCNA analysis demonstrated that DEGs and DELs are primarily divided into three main modules (cyan module, darkslateblue module and paleturquoise module). Core gene analysis within these modules identified *PKD2*, *SOX13* and *COL5A1* are implicated in adipose tissue development or adipocyte differentiation, and all three genes are target genes of lncRNA within the network. However, further research is needed to explore the impact of genes including *PAK2*, *LARS*, *BIRC6*, *PKD2*, *SOX13*, *PITPNM2*, *COL5A1*, *OLFML2A* and *P3H3* on IMF in porcine LDM, as well as the effects of candidate lncRNAs MSTRG.21592.2, MSTRG.8859.2, MSTRG.18175.1, MSTRG.9969.1 and MSTRG.180.2 on meat quality via molecular regulation.

## Data Availability

The datasets presented in this study can be found in online repositories. The names of the repository/repositories and accession number(s) can be found at: https://www.ncbi.nlm.nih.gov/bioproject/, PRJNA721288.

## Footnotes

^1^https://github.com/najoshi/sickle

^2^https://github.com/jstjohn/SeqPrep
